# Plasma surfactant protein-A levels in apparently healthy smokers, stable and exacerbation COPD patients

**DOI:** 10.12669/pjms.344.13951

**Published:** 2018

**Authors:** Nida, Khalid Parvez Lone

**Affiliations:** 1 Nida, Department of Physiology & Cell Biology, The University of Health Sciences, Khayaban-e-Jamia, Lahore, Pakistan; 2 Khalid Parvez Lone, Department of Physiology & Cell Biology, The University of Health Sciences, Khayaban-e-Jamia, Lahore, Pakistan

**Keywords:** BMI, COPD, ELISA, FEV, FEV1, Surfactant protein A

## Abstract

**Objective:**

To compare the plasma level of surfactant protein-A in apparently healthy smokers, stable and exacerbation Chronic Obstructive Pulmonary Disease (COPD) patients.

**Methods:**

This was a comparative study conducted from January, 2015 to March, 2016. This study was conducted on 87 subjects of both gender and age between 30-70 years. Of the total 87 subjects; 29 subjects were “healthy smokers” selected from general population as control group. Another 29 were “stable COPD” patients free of exacerbation since last six weeks. Lastly, another 29 subjects were “exacerbated COPD” patients with 7-10 days of exacerbation. COPD was diagnosed on the basis of relevant history and spirometry showing post bronchodilator FEV1/FVC <0.70. Surfactant Protein-A level (ng/ml) was estimated by a specific solid phase enzyme linked immunosorbent assay (ELISA) using automated EIA analyzer.

**Results:**

The SP-A levels, determined by competitive ELISA, was significantly higher (P<0.025) in healthy smokers (44.19±39.17 ng/ml) and exacerbated (43.86±40.17) than the stable COPD (25.89±18.85) patients. The lung function parameters (FEV1, FVC and FEV1/ FVC) were lower in COPD patients compared to healthy smokers and were related to the duration of smoking.

**Conclusion:**

Current smokers and exacerbated patients had higher values of SP-A protein than stable COPD patients since they had stopped smoking.

## INTRODUCTION

Despite various government actions against it, smoking is a community based issue and its prevalence is continuously increasing. According to World Health Organization (WHO), smoking is a factor which kills one in ten adults, globally.^1^ Smoking at an early age is most likely to develop in to chronic condition such as “Chronic Obstructive Pulmonary Disease (COPD)”.^2^ According to GOLD criteria, COPD is defined as, “It is a systemic disease characterized by airflow limitation that is irreversible and progressive and is associated with an abnormal inflammatory responses of the lungs to noxious particles or gases”.^3^ The economic burden due to COPD is increasing in United States with a prevalence of about 7.6% - 8.9%.^4^ Since, in the early stage of the disease, there are no significant clinical features; the total burden of COPD is therefore, underestimated.

The main cause of COPD is a long-term exposure to inhaled toxins and gases. Among them, cigarette smoke is on the top and causes more than 90% of the cases reported in both developing and developed countries.^5^ Surfactant is a thin layer, lining the alveoli and terminal bronchioles, and it is a mixture of lipids and proteins synthesized mainly by Type-II pneumocytes. There are four types of surfactant proteins namely “surfactant protein– A (SP-A)”, “surfactant protein–B (SP-B)”, “surfactant protein–C (SP-C)” and “Surfactant protein– D (SP-D)”.^6^ Among them, the most abundant surfactant protein is “surfactant protein –A”.^7^ SP-A maintains the lungs innate immunity by protecting the lungs against allergens and bacteria.^8^ There is a dearth of lung specific biomarkers causing limitation in diagnosis of COPD and development of novel therapies. Therefore, there is a necessity for a simple biomarker to be utilized in clinical settings.^9^ Surfactant Protein –A, a lung – specific secretory protein fit this scenario well. It can be a diagnostic marker for non-invasive assessment of lung epithelium function.^10^

No previous study has been done to highlight the role of surfactant protein A (SP-A) in COPD in the local population. Spirometry is seldom used to assess lung function and most of the clinicians rely on bedside techniques to determine the progress of the disease and its control. There is a lack of epidemiological data regarding the normal values of SP-A in the local population, which is found to be different than those of the western world owing to different ethnic, social, geographical and environmental, factors.^11^ The present study will help establish a base line for SP-A in local smokers and COPD patients and results may help us to understand its role in disease. SP-A may become a reliable biomarker for COPD in future. Based on the available literatures and non-availability of data regarding local population, the present study was designed to determine and compare the SP-A levels in healthy smokers, stable COPD patients and exacerbated COPD patients.

## METHODS

This was a comparative study conducted from January, 2015 to March, 2016 at Department of Physiology and Cell Biology, University of Health Sciences Lahore, Pakistan, after approval from Institutional Review Board and Ethical Research Committee.

### Sample size calculation

With 80% power of study, 5% level of significance and use of mean and standard deviation from literature, a sample size of 29 in each group and a total of 87 subjects were used. The following formula was used for calculating the number of samples required per group.


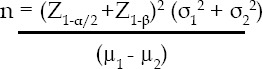


***Where:***

**Z1-β** is the desired power of study = 80% (0.84), **Z1-α/2** is the desired level of significance = 5% (1.96)

**µ1 - µ2** Mean difference = 76, **σ1** = 83, **σ2** = 120; therefore **n=** 29/group

This study included a total 87 subjects of both sexes (male and female). The subject age group was between 30-70 years and out of the total 87; a group of 29 subjects were “healthy smokers” selected from the general population and were taken as the control group and defined as “smokers with a normal pulmonary function with cellular and structural changes occurring in lungs however, there is no airflow limitation”. Another 29 subjects were “stable chronic obstructive pulmonary disease (COPD)” patients that were free of exacerbation since last six weeks and were selected from the chronic obstructive pulmonary disease (COPD) clinic at Gulab Devi Chest Hospital, Lahore and defined as “A disease caused by poorly reversible airflow limitation which causes abnormal inflammatory response of the lung and is being progressive”.^12^ Lastly, another 29 subjects were “Acute exacerbated chronic obstructive pulmonary disease (ACOPD)” patients with 7-10 days of exacerbation and selected from the COPD emergency department at Gulab Devi Chest Hospital, Lahore and defined as “A condition in which there is a worsening of symptoms that causes a significant impact on health of patient which results in progressive decline in lung function”.^13^

After recording required demographic details and using standardized techniques anthropometric measurements like height, weight and waist circumference (WC) were recorded. Before anthropometry, participants were guided to remove ornaments, jewelry, shoes, wrist watch, mobile phone and wallets from their pockets.^14^ Spirometry was done to assess the lungs functions. According to the American Thoracic Society (ATS) criteria, spirogram was obtained from all participants.^15^ Pulmonary function parameters were assessed by using flow–measuring type spirometer called “Spirolab II, bidirectional digital turbine type spirometer” (Medical International Research, Rome, Italy). Spirometer has a turbine sensor and a mouth piece is needed to connect a participant to the spirometer. During spirometry, there is “Forced expiratory maneuver” which is defined as the person exhales completely and forcefully from a point of maximum inhalation.^16^

Spirometry was done according to the “GOLD” (Global Initiative for Chronic Obstructive Lung Disease) standard with 400µg salbutamol. The spirometric test was repeated after 20 minutes.^17^ It is very essential to assess FEV1 and FVC for all smokers and ex-smokers and when FEV1 / FVC is <70% predicted, and FEV1 is <80% predicted, smokers have COPD.^18^

For spirometry, the participant was comfortably seated on a chair and five inches above the elbow joint tourniquet was applied. Five milliliter of blood was drawn from the antecubital vein in an EDTA vacutainer. Plasma was separated by centrifugation at 15,000 rpm for 10 minutes and was stored at -80°C until analyzed, in separate labeled aliquots. Surfactant Protein A-level (ng/ml) was estimated by solid phase enzyme linked immunosorbent assay (ELISA) using automated EIA analyzer (Bio-Rad, USA with commercially available kit by Glory Science, TX, USA).

### Statistical analysis

Data were analyzed using the SPSS (Version 20). Shapiro-Wilk test was used to assess the normality of the data. If data were normally distributed, mean ± SD (standard deviation) were given. If the data were non-normally distributed, median with IQR (interquartile ratio) were used. For normally distributed data, One Way ANOVA with Post-hoc Tukey’s test was used. For non-normally distributed data, Kruskal-Wallis test was applied and Mann Whitney-U test was used as post hoc test. A p ≤ 0.05 was considered as statistically significant.

## RESULTS

As this study used non-probability convenient sampling, the subjects were comprised of 8% females and 92% males. Out of the total 87 subjects, 65 were current smokers and 22 were ex-smokers. This study comprised of three experimental groups, that is, “Healthy smokers”, “Stable COPD patients” and “Exacerbated COPD patients”. The current and ex-smokers were present in all the three groups. Out of the 65 current smokers, 23 subjects were in healthy smokers group, 20 in stable COPD group and 22 subjects were in exacerbated COPD group. Similarly, out of the 22 ex-smokers, six subjects were in healthy smokers, nine in stable COPD group and seven subjects were in exacerbated COPD group. There was a statistically significant difference in the age, weight and BMI of the study subjects in the three groups, while the height was not statistically significant (Table-I).

**Table-I:** Comparison of anthropometry parameters, lung function tests and SP-A levels between healthy smokers, stable COPD and exacerbated COPD groups.

Parameter	Healthy Smokers	Stable (COPD)	Exacerbated (COPD)	p-value

	Mean ± SD / Median (IQR)^a^	Mean ± SD / Median (IQR)^a^	Mean ± SD / Median (IQR)^a^	
Age^a^ (years)	48.34±8.39	53.14±14.37	57.31±16.24	0.044^*b^
Weight^a^ (kg)	71.55±14.39	54.00 (45.00-69.50)	60.00 (46.50-74.00)	0.003^*c^
Height^a^ (cm)	169.06±8.87	166.75±8.03	169.20±5.89	0.401^b^
BMI^a^ (kg/m^2^)	24.92 ± 4.04	20.82±4.29	19.15 (16.78-25.81)	0.002^*c^
Measured FEV_1_^a^ (liters)	3.05±0.57	1.30 (1.10-1.85)	1.03±0.34	<0.001^*c^
Measured FVC^a^ (liters)	3.84 (3.18-4.24)	2.03 (1.73-3.05)	1.68 (1.46-2.51)	<0.001^*c^
Measured^a^ FEV_1_/FVC (%)	81.71±4.73	60.81±6.30	54.40±8.18	<0.001^*b^
Surfactant^a^ Protein A (ng/ml)	34.88 (26.70-51.30)	25.89±18.85	35.94 (26.01-47.34)	0.025^*c^

a Values are given as mean ± SD for normally distributed variables and median (IQR) for non-normally distributed variables,

b p-value is generated by One Way ANOVA,

c p-value is generated by Kruskal-Wallis test,

* p-value ≤ 0.05 is considered statistically significant

The difference in the lung function parameters such as FEV_1_, FVC and FEV_1_ /FVC ratio was statistically different in the three study groups (P<0.001). Similarly plasma surfactant protein A levels were also significantly (*P*<0.05) different in the study groups (Table-I).

The difference in the mean FEV_1_and FVC in the study groups was statistically significant (*P=*0.046 and 0.048, respectively) among the currents smokers and ex-smokers. However, ratio of FEV_1_ / FVC was not statistically significant (*P=*0.446) among these two groups (Table-II).

**Table-II:** Comparison of Lung Function Tests between current and ex-smokers used in the present study irrespective of the presence or absence of COPD.

Sr. No.	Parameter	Current smokers (n = 65)	Ex-smokers (n = 22)	p-value

		Mean ± SD / Median (IQR)^a^	Mean ± SD / Median (IQR)^a^	
1	Measured FEV_1_ (liters)	1.57 (1.12-2.79)	1.09 (0.90-2.51)	0.046^*b^
2	Measured FVC (liters)	2.82 (1.78-3.77)	2.28±0.99	0.048^*b^
3	Measured FEV_1_/FVC (%)	64.88 (55.63-78.73)	63.86±11.64	0.446^b^

a Values are given as mean ± SD for normally distributed variables and median (IQR) for non-normally distributed variables,

b p-value is generated by Mann Whitney-U test

* p-value ≤ 0.05 is considered statistically significant.

## DISCUSSION

The results of the present study show that increasing age is one of the risk factors for COPD because cumulative effects of smoking and exposure to other pollutants increase with age. In the present study, the highest age found in exacerbated COPD patients was (57.31±16.24 years) followed by stable COPD patients (53.14±14.37), while the minimum age (48.34±8.39) was encountered in asymptomatic chronic smokers (Table-I). This observation regarding age is consistent with earlier data pointing to the fact that with increasing age, gas trapping, increase in airspace abnormalities and tobacco smoke aggravates these changes.^10^

The numbers of males in this study were greater than that of females in COPD groups. The males were 92% and the females were only 8%. These are striking differences between the sexes in prevalence of COPD. Male sex is identified as a risk factor for COPD and there is increased prevalence of smoking amongst males in Indian subcontinent, including Pakistan.^19^ This finding is consistent with the present study. Although this may not be a rule in other countries. Danish females were more susceptible to the adverse effects of smoking on lung function. This may also point to the fact that smoking is quite prevalent in females of Western society.^20^

The pulmonary function parameters were found to be significantly different among the study groups. The FEV_1_/FVC ratio was associated with the increase in the disease, in as much as, the highest values were encountered in chronic smokers with no disease to stable COPD patients, with the minimum values seen in exacerbated COPD patients (Table-I). This finding is consistent with the earlier findings that these spirometric variables decline as the disease progresses.^21^

Lung surfactant is a mixture of proteins and lipids that cover the alveolar epithelial surface and is necessary for normal lung function. SP-A, located on the lung surface, is directly exposed to cigarette smoke.^22^ SP-A level is increased in blood of smokers because SP-A being a lung protein leaks during pulmonary inflammation in to the blood. In the present study the levels of SP-A were comparable in healthy smokers (range= 26.70-51.30 ng/ml) to exacerbated COPD patients (26.01-47.34 ng/ml) and there was no significant difference between these two groups. However; the blood levels of this protein in stable COPD patients were significantly lower than the two other groups (Table-I).

In healthy current smokers, the airways and lung parenchyma generally show inflammatory changes and structural abnormalities due to cigarette smoke thus leaking the SP-A in to the blood.^23^ In the present study, SP-A levels were comparable with the levels found in a Japan study which also showed that, SP-A levels in smokers (29.8±15.7 ng/ml) was significantly higher than in non-smokers. The normal range of SP-A in healthy non-smokers was 18.4±8.5 ng/ml.^7^ SP-A level is increased in blood of smokers because SP-A is a lung protein and during pulmonary inflammation SP-A leaks in to the blood because SP-A, located on the lung surface, is directly exposed to cigarette smoke.^19^ Our findings were also supported by another study conducted in India showing that the SP-A levels in smokers (54.2±42.98 ng/ml) were significantly higher than in nonsmokers (3.62±1.86 ng/ml).^10^

In the present study, the values of SP-A were higher in healthy smokers than the stable COPD patients and were comparable to the exacerbated COPD patients. The reason for this effect is that the healthy smokers group was still smoking as compared with the stable COPD patients who had nearly left the habit of smoking for variable duration. That exacerbated COPD patients had values comparable to healthy smokers and higher than the stable COPD patients is due to the fact that exacerbation and severity of diseases had increased the pulmonary inflammation and thus the level of SP-A in the blood.^24^ These results points to the fact that these values could be used as a screening test to differentiate between smokers and non-smokers and stable COPD and exacerbated COPD patients. However, further detailed studies with much higher subject numbers are needed to generalize this observation.

There is a strong relationship between the amount and duration of smoking with increasing risk of COPD. The person who smokes more has increased chances of having airway epithelial permeability. Around the world; the graph of tobacco smoking has paralleled to increase in COPD incidence including India and Pakistan.^19^ In this study, there was least number of current smokers in stable COPD patients compared to other two groups. As reported above, cigarette smoke causes destruction of connective tissue in the alveoli and leads to increased lung epithelial permeability and increases the leakage of SP-A in to blood^22^ and this effect would be further enhanced in current smokers. Smoking is the most important causative factor in the development of COPD. In this regard, findings from India are in line with the result of present study in that the higher the degree of smoking intensity, the greater is the decline in FEV1.^19^

### Limitations

The sample size of the study was not large enough to validate these results in the entire population. The sampling technique was purposive sampling, so it is difficult to generalize the finding beyond the sample. Also, this study was not a longitudinal one; therefore, we could not keep the subjects under observation over a long period of time to evaluate the level of SP-A in smokers, ex-smokers and their lung function. Studies are needed in all these directions.

## CONCLUSIONS

There is a lower level of plasma SP-A in stable COPD as compared with exacerbated COPD and healthy smokers presenting at the COPD clinic of Gulab Devi Chest Hospital, Lahore. SP-A levels may be used for screening, in future, between smokers, non-smokers and patients of stable COPD who have left smoking and exacerbated patients. There is a decline in lung function in the all COPD subjects, whether stable or exacerbated, as compared to healthy smokers.

### Authors’ Contribution

**N**: Collection of data and statistical analysis, Spirometry, drafting the article.

**KPL:** Conception and design, revising the article critically for important intellectual content, Analysis and interpretation of data, and Final version of the Manuscript.

## References

[1] Raza MZ, Ahmed A, Ahmed F, Ghani A, Rizvi N (2013). COPD exacerbations:epidemiology and impact on patient's outcome. Int J Environ Sci.

[2] Jawed S, Ejaz S, Rehman R (2012). Influence of smoking on lung functions in young adults. J Pak Med Asoc.

[3] Pauwels RA, Buist AS, Calverley PM, Jenkins CR, Hurd SS (2001). Global strategy for the diagnosis, management, and prevention of chronic obstructive pulmonary disease. NHLBI/WHO Global Initiative for Chronic Obstructive Lung Disease (GOLD) Workshop Summary. Am J Respir Crit Care Med.

[4] Csikesz NG, Gartman EJ (2014). New developments in the assessment of COPD:early diagnosis is key. Int J Chron Obstruct Pulmon Dis.

[5] Brashier BB, Kodgule R (2012). Risk factors and pathophysiology of chronic obstructive pulmonary disease (COPD). J Assoc Physicians India.

[6] Pastva AM, Wright JR, Williams KL (2007). Immunomodulatory roles of surfactant proteins A and D:implications in lung disease. Proc Am Thorac Soc.

[7] Nomori H, Horio H, Fuyuno G, Kobayashi R, Morinaga S, Suemasu K (1998). Serum surfactant protein A levels in healthy individuals are increased in smokers. Lung.

[8] Mazur W, Tolijamo T, Ohlmeier S, Vuopala K, Nieminen P, Kobayashi H (2011). Elevation of surfactant protein A in plasma and sputum in cigarette smokers. Eur Respir J.

[9] Cazzola M, MacNaee W, Martinez FJ, Rabe KF, Franciosi LG, Barnes PJ (2008). Outcomes for COPD pharmacological trials:from lung function to biomarkers. Eur Respir J.

[10] Behera D, Balamugesh T, Venkateswarlu D, Gupta A, Majumdar S (2005). Serum surfactant protein A levels in chronic bronchitis and its relation to smoking. Indian J Chest Dis Allied Sci.

[11] Bhandari R, Sharma R (2012). Epidemiology of chronic obstructive pulmonary disease:a descriptive study in the mid- western region of Nepal. Int J Chron Obstruct Pulmon Dis.

[12] Saetta M, Turato G, Maestrelli P, Mapp CE, Fabbri LM (2001). Cellular and structural bases of chronic obstructive pulmonary disease. Am J Respir Crit Care Med.

[13] Donaldson GC, Wedzicha JA (2013). Deprivation, winter season, and COPD exacerbations. Prim Care Respir J.

[14] Sharp C (1996). Kinanthropometry and Exercise Physiology Laboratory Manual. Tests, procedures and data. Br J Sports Med.

[15] Miller MR, Hankinson J, Brusasco V, Burgos F, Casaburi R, Coates A (2005). Standardization of spirometry. Eur Respir J.

[16] Joo MJ, Au DH, Fitzgibbon ML, McKell J, Lee TA (2011). Determinants of spirometry use and accuracy of COPD diagnosis in primary care. J Gen Intern Med.

[17] Erdogan A, Ucar EY, Araz O, Saglam L, Mirici NA (2013). Contribution of spirometry to early diagnosis of chronic obstructive pulmonary disease in primary health care centers. Turk J Med Sci.

[18] Briggs DD (2004). Chronic Obstructive Pulmonary Disease Overview:Prevalence, Pathogenesis, and Treatment. J Manag Care Pharm.

[19] Jindal SK (2006). Emergence of chronic obstructive pulmonary disease as an epidemic in India. Indian J Med Res.

[20] Prescott E, Bjerg AM, Andersen PK, Lange P, Vestbo J (1997). Gender difference in smoking effects on lung function and risk of hospitalization for COPD:result from a Danish longitudinal population study. Eur Respir J.

[21] Rabe KF, Fabbri LM, Vogelmeier C, Kogler H, Schmidt H, Beeh KM (2013). Seasonal distribution of COPD Exacerbations in the prevention of exacerbations with tiotropium in COPD trial. Chest.

[22] Jin H, Webb-Robertson BJ, Peterson ES, Tan R, Bigelow DJ, Scholand MB (2011). Smoking, COPD, and 3-Nitrotyrosine levels of plasma proteins. Environ Health Perspect.

[23] Hogg JC (2004). Pathophysiology of airflow limitation in chronic obstructive pulmonary disease. Lancet.

[24] Ilumets H, Mazur W, Toljamo T, Louhelainen N, Nieminen P, Kobayashi H (2011). Ageing and smoking contribute to plasma surfactant proteins and protease imbalance with correlations to airway obstruction. BMC Pulmonary Med.

[25] Shakoori TA, Sin DD, Ghafoor F, Bashir S, Bokhari HNS (2009). Serum surfactant protein D during acute exacerbations of chronic obstructive pulmonary disease. Dis Marker.

